# Binding and Signaling Studies Disclose a Potential Allosteric Site for Cannabidiol in Cannabinoid CB_2_ Receptors

**DOI:** 10.3389/fphar.2017.00744

**Published:** 2017-10-23

**Authors:** Eva Martínez-Pinilla, Katia Varani, Irene Reyes-Resina, Edgar Angelats, Fabrizio Vincenzi, Carlos Ferreiro-Vera, Julen Oyarzabal, Enric I. Canela, José L. Lanciego, Xavier Nadal, Gemma Navarro, Pier Andrea Borea, Rafael Franco

**Affiliations:** ^1^Instituto de Neurociencias del Principado de Asturias, Departamento de Morfología y Biología Celular, Facultad de Medicina, Universidad de Oviedo, Asturias, Spain; ^2^Pharmacology Institute, Department of Medical Sciences, University of Ferrara, Ferrara, Italy; ^3^Molecular Neurobiology Laboratory, Department of Biochemistry and Molecular Biomedicine, University of Barcelona, Barcelona, Spain; ^4^Centro de Investigación Biomédica en Red Enfermedades Neurodegenerativas, Instituto de Salud Carlos III, Madrid, Spain; ^5^Phytoplant Research S.L., Córdoba, Spain; ^6^Small Molecule Discovery Platform, Molecular Therapeutics Program, Center for Applied Medical Research, University of Navarra, Pamplona, Spain; ^7^Neuroscience Department, Center for Applied Medical Research, University of Navarra, Pamplona, Spain; ^8^Department of Biochemistry and Physiology, Faculty of Pharmacy, University of Barcelona, Barcelona, Spain

**Keywords:** endocannabinoid, allosterism, G-protein-coupled receptor, phytocannabinoids, SR144528, irreversible, TR-FRET

## Abstract

The mechanism of action of cannabidiol (CBD), the main non-psychotropic component of *Cannabis sativa* L., is not completely understood. First assumed that the compound was acting via cannabinoid CB_2_ receptors (CB_2_Rs) it is now suggested that it interacts with non-cannabinoid G-protein-coupled receptors (GPCRs); however, CBD does not bind with high affinity to the orthosteric site of any GPCR. To search for alternative explanations, we tested CBD as a potential allosteric ligand of CB_2_R. Radioligand and non-radioactive homogeneous binding, intracellular cAMP determination and ERK1/2 phosphorylation assays were undertaken in heterologous systems expressing the human version of CB_2_R. Using membrane preparations from CB_2_R-expressing HEK-293T (human embryonic kidney 293T) cells, we confirmed that CBD does not bind with high affinity to the orthosteric site of the human CB_2_R where the synthetic cannabinoid, [^3^H]-WIN 55,212-2, binds. CBD was, however, able to produce minor but consistent reduction in the homogeneous binding assays in living cells using the fluorophore-conjugated CB_2_R-selective compound, CM-157. The effect on binding to CB_2_R-expressing living cells was different to that exerted by the orthosteric antagonist, SR144528, which decreased the maximum binding without changing the *K_D_*. CBD at nanomolar concentrations was also able to significantly reduce the effect of the selective CB_2_R agonist, JWH133, on forskolin-induced intracellular cAMP levels and on activation of the MAP kinase pathway. These results may help to understand CBD mode of action and may serve to revisit its therapeutic possibilities.

## Introduction

The endocannabinoid system consists of two compounds with amphipathic structure, 2-arachidonyl glycerol (2-AG) and anandamide (AEA), the enzymes that produce and degrade them, and two cannabinoid, CB_1_ and CB_2_, receptors (see Lu and MacKie, 2016 and references therein). Recent evidence presents the endocannabinoid system as one of the most relevant in mammals for homeostatic control of energy expenditure and temperature, and for regulating innate and acquired immunity and neural transmission. Cannabinoid receptors belong to the superfamily of G-protein-coupled receptors (GPCRs) and are reportedly coupled to the heterotrimeric G_i_ protein^1^, i.e., their activation leads to a decrease in the intracellular level of a second messenger, cAMP. Although CB_1_ receptors (CB_1_Rs) are mainly located in neurons of the central nervous system (CNS), their expression in glia has been described (Stella, 2010; Bilkei-Gorzo, 2012). In fact, CB_1_Rs are considered to be the most abundant GPCRs in the CNS and the targets of the Δ^9^-tetrahydrocannabinol (Δ^9^-THC), the main psychotropic component of *Cannabis sativa* L. CB_2_ receptor (CB_2_R), which soon after its identification was considered a prototypic peripheral receptor, is also expressed in the CNS, both in glia (Zhang et al., 2003; Cabral and Marciano-Cabral, 2005; Fernández-Ruiz et al., 2007) and neurons of some specific brain regions such as cerebellum or *globus pallidus* (Lanciego et al., 2011; Sierra et al., 2015). Heteromerization of CB_1_R/CB_2_R in the brain is a well-accepted phenomenon that further diversifies the physiological actions of endo- and exogenous cannabinoids (Callén et al., 2012; Sierra et al., 2015). Although physiological relevance of this heteromer expression is not fully understood, evidence suggests potential in CNS-related drug discovery.

Apart from heteromerization, drug discovery efforts for cannabinoid receptors also involve the use of allosteric cannabinoid ligands. Whereas cannabinoids interact with the orthosteric receptor site, allosteric modulators bind to a topographically distinct binding site modifying receptor conformation and leading to novel properties and modes of action (Conn et al., 2009). In fact, allosteric modulators can impact on the affinity of the orthosteric binding pocket and also in the intracellular signaling responses, in either a positive or negative direction. To date, a number of allosteric cannabinoid receptor modulators have been reported for CB_1_R, e.g., lipoxin A4, ORG27569, PSNCBAM-1 or cannabidiol (CBD) (Pamplona et al., 2012; Laprairie et al., 2015; Morales et al., 2016).

CBD, the main non-psychoactive phytocannabinoid, exerts a wide range of cellular effects through the endocannabinoid system, as was indicated in a systematic review in 2015 (McPartland et al., 2015). However, its mode of action is far from being understood. In some *in vitro* assays, CBD action has been linked to CB_2_Rs activation because some *in vivo* effects were blocked by the administration of a CB_2_R antagonist. Thus, Ignatowska-Jankowska et al. (2011), using AM630 as antagonist, reported that the weight gain-reducing effect of CBD apparently involved CB_2_Rs. In addition, the well-substantiated protective role of CBD in newborn hypoxia-ischemia correlates with CB_2_R expression (Castillo et al., 2007; Alvarez et al., 2008; Lafuente et al., 2011; Pazos et al., 2013). In other *in vitro* assays, CBD was shown as a high potency antagonist of CB_2_R expressed in CHO cells (Thomas et al., 2009). Nevertheless, radioligand and/or GTPgammaS binding assays indicate that the compound does not bind with high affinity to either rat or human CB_2_Rs (Hanus et al., 2005; Pertwee, 2008; McPartland et al., 2009). At present, the idea that CBD actions are mediated by binding to serotonin 5-HT_1A_ receptors is favored. For instance, CBD affords protection against oxygen-glucose deprivation in a model of the blood–brain barrier by a mechanism involving 5-HT_1A_ and PPARγ receptors (Hind et al., 2016). Further behavioral studies also suggest that CBD may act via 5-HT_1A_ receptors (Russo et al., 2005; Alves et al., 2010; Magen et al., 2010; Rock et al., 2012; Espejo-Porras et al., 2013; Sartim et al., 2016). Such assumption is intriguing as the reported affinity of CBD binding to these serotonin receptors does not lie within the nanomolar high affinity range (Russo et al., 2005). More recently, some authors open new avenues in the therapeutic use of this phytocannabinoid. Thus, they seem to demonstrate that CBD was able to reduce the effect of 2-AG and Δ^9^-THC on CB_1_R internalization and PLCβ3 and ERK1/2 phosphorylation, proposing that CBD may act as a negative allosteric modulator of these receptors (Laprairie et al., 2015).

The aim of this work was to search for the possibility that CBD acts as an allosteric ligand of CB_2_R by checking whether it is able to modulate the binding and functional effect of CB_2_R agonists.

## Materials and Methods

### Materials and Reagents

For radioligand binding assays, [^3^H]-(R)-(+)-[2,3-dihydro-5-methyl-3-(4-morpholinyl methyl)pyrrolo[1,2,3-de]-1,4-benzoxazin-6-yl]-1-naphthalenylmethanone ([^3^H]-WIN 55,212-2) was purchased from PerkinElmer (Wellesley, MA, United States). CBD, WIN 55,212-2 mesylate and SR144528 were obtained from Tocris Bioscience (Bristol, United Kingdom). THC was obtained from THC Pharm (Frankfurt, Germany).

For non-radioactive binding assays, the Tag-lite labeling medium (TLB) was obtained from Cisbio Bioassays (LABMED, Cisbio Assays, Codolet, France). The Tb derivative of O6-benzylguanine was synthesized by Cisbio Bioassays and is commercialized as SNAP-Lumi4-Tb (SSNPTBC, Cisbio Assays, Codolet, France). CB_2_R agonist, 3-[[4-[2-*tert*-butyl-1-(tetrahydropyran-4-ylmethyl)benzimidazol-5-yl]sulfonyl-2-pyridyl]oxy]propan-1-amine (CM-157), conjugated to red-naltrexone fluorescent probe (red CB_2_R ligand) was developed by Cisbio Bioassays. The unlabeled compound was synthesized as described in Martínez-Pinilla et al. (2016) based on the information given in the WO2008003665 patent and in Verbist et al. (2008). Stock solutions were prepared in DMSO. Aliquots of these stock solutions were kept frozen at -20°C until use. The plasmid encoding for the SNAP-tagged human CB_2_R used for transient transfection was obtained from Cisbio Bioassays (PSNAP-CB2, Cisbio Assays, Codolet, France). The white opaque 384-well plates were obtained from PerkinElmer (Wellesley, MA, United States).

### Radioligand Binding Assays

#### Cell Culture and Membrane Preparation

CHO cells stably expressing human CB_2_R (CHO-CB_2_R) (PerkinElmer, United States) were grown adherently and maintained in Ham’s F12 medium containing 10% heat-inactivated fetal bovine serum (FBS), 100 units/mL penicillin/streptomycin and 0.4 mg/mL geneticin (G418) at 37°C in 5% CO_2_ humid atmosphere.

For membrane isolation, culture medium was removed and cells were washed with phosphate-buffered saline (PBS) and scraped off in ice-cold hypotonic buffer (5 mM Tris–HCl, 2 mM EDTA, pH 7.4). The cell suspension was homogenized with a Polytron and then centrifuged for 30 min at 40,000 × *g*. The membrane pellet was suspended in 50 mM Tris–HCl buffer (pH 7.4) containing 1 mM EDTA, 5 mM MgCl_2_, 0.5 mg/mL bovine serum albumin (BSA).

#### Saturation Binding Experiments

[^3^H]-WIN 55,212-2 saturation binding experiments (specific activity 48 Ci/mmol, PerkinElmer) were performed incubating different concentrations of the radioligand (0.2–40 nM) in binding buffer (50 mM Tris–HCl, pH 7.4, 1 mM EDTA, 5 mM MgCl_2_) with membranes from CHO cells stably expressing the human CB_2_R (10 μg per sample) at 30°C. Non-specific binding was determined in the presence of 1 μM WIN 55,212-2. At the end of the incubation period (60 min) bound and free radioactivity were separated in a Brandel cell harvester (Brandel Instruments) by filtering the assay mixture through Whatman GF/B glass fiber filters. The filter-bound radioactivity was counted using a Packard Tri Carb 2810 TR scintillation counter (PerkinElmer, Wellesley, MA, United States).

#### Association Binding Experiments

Association binding experiments for [^3^H]-WIN 55,212-2 were performed incubating three different concentrations (0.8, 1.6, and 3.2 nM) of the radioligand in binding buffer (50 mM Tris–HCl, pH 7.4, 1 mM EDTA, 5 mM MgCl_2_) with human CHO-CB_2_R cell membranes at 30°C. Non-specific binding was determined in the presence of 1 μM WIN 55,212-2. Free and bound radioligand were separated through filtration at multiple time points (from 1 to 50 min) to construct association kinetic curves.

#### Dissociation Binding Experiments

The dissociation rate of [^3^H]-WIN 55,212-2 was determined by allowing 3 nM radioligand to reach equilibrium with human CHO-CB_2_R cell membranes at 30°C for 60 min. To start dissociation, WIN 55,212-2 (10 μM final concentration) was added in order to occupy binding sites as they became available, thereby preventing re-association. The amount of radioactivity that remained bound to the receptor was determined by filtration harvesting and scintillation counting at different time points (from 0 to 50 min).

#### Competition Binding Experiments

Competition binding experiments were performed incubating 3 nM [^3^H]-WIN 55,212-2 and different concentrations of the tested compounds with membranes obtained from CHO-CB_2_R cells (10 μg protein per sample) for 60 min at 30°C. Non-specific binding was determined in the presence of 1 μM WIN 55,212-2. Bound and free radioactivity were separated by filtering the assay mixture through Whatman GF/B glass fiber filters using a Brandel cell harvester. The filter-bound radioactivity was counted using a Packard Tri Carb 2810 TR scintillation counter (PerkinElmer).

### Non-radioligand Binding Assays

#### Cell Line Cultures and Transfection

Human embryonic kidney 293T (HEK-293T) cells were grown in DMEM supplemented with 2 mM L-glutamine, 1 mM sodium pyruvate, 100 units/mL penicillin/streptomycin, and 5% (v/v) FBS (all supplements were from Invitrogen, Paisley, Scotland, United Kingdom). Cells were maintained at 37°C in a humidified atmosphere of 5% CO_2_ and were passaged, with enzyme-free cell dissociation buffer (13151-014, Gibco^®^, Thermo Fisher Scientific, Waltham, MA, United States), when they were 80–90% confluent, i.e., approximately twice a week.

For fluorescent ligand-binding assays, HEK-293T cells growing in 25-cm^2^ flasks were transiently transfected with the SNAP-CB_2_R plasmid cloned in pcDNA3.1 by the Lipofectamine 2000 method (11668-019, Invitrogen, Thermo Fisher Scientific, Waltham, MA, United States). When reaching 60% confluence, cell medium was removed and replaced by 4 mL of fresh medium. In parallel, a transfection mix containing 8 μg of plasmid, 20 μL of Lipofectamine 2000, and 1 mL of Opti-MEM without serum (51985-026, Gibco^®^, Thermo Fisher Scientific, Waltham, MA, United States) final volume was incubated for 20 min at room temperature prior to being added on cells. The transfected-cell culture flask was incubated at 37°C under 5% CO_2_ for 24 h.

#### Labeling of Cells Expressing SNAP-Tagged CB_2_R

Cell culture medium was removed from the 25-cm^2^ flask and 100 nM SNAP-Lumi4-Tb, previously diluted in 3 mL of TLB 1×, was added to the flask and incubated for 1 h at 37°C under 5% CO_2_ atmosphere in a cell incubator. After that, cells were washed four times with 2 mL of TLB 1× to remove the excess of SNAP-Lumi4-Tb, detached with enzyme-free cell dissociation buffer, centrifuged 5 min at 1,500 rpm and collected in 1 mL of TLB 1×. Tag-lite-based binding assays were performed 24 h after transfection. Densities from 2,500 to 3,000 cells per well were used to carry out binding assays in suspension in white opaque 384-well plates.

#### Competition and Saturation Binding Assays

For competition binding assay, red CB_2_R ligand (labeled CM-157) and CBD were diluted in TLB 1×. HEK-293T cells transiently expressing Tb-labeled SNAP-CB_2_R were incubated with 100 nM red CB_2_R ligand, in the presence of increasing concentrations (0–10 μM range) of CBD. In plates containing 10 μL of labeled cells, 5 μL of TLB 1× or 5 μL of CBD was added prior to the addition of 5 μL of fluorescent ligand. Plates were then incubated for at least 2 h at room temperature before signal detection.

Saturation binding experiments were performed by incubating HEK-293T cells transiently expressing Tb-labeled SNAP-CB_2_R with increasing concentrations of the red CB_2_R ligand (range, 0–300 nM final) in TLB 1×. For each concentration, non-specific binding was determined by adding 100 μM unlabeled CB_2_R ligand. In the plates containing 10 μL labeled cells, 5 μL of 100 μM unlabeled CB_2_R ligand or TLB 1× was added, followed by the addition of 5 μL of increasing concentrations of the red CB_2_R ligand. Plates were incubated for 2 h at room temperature before signal reading.

Signal was detected using an EnVision microplate reader (PerkinElmer, Waltham, MA, United States) equipped with a FRET optic module allowing donor excitation at 337 nm and signal collection at both 665 and 620 nm. A frequency of 10 flashes/well was selected for the xenon flash lamp excitation. The signal was collected at both 665 and 620 nm using the following time-resolved settings: delay, 150 μs; integration time, 500 μs. HTRF ratios were obtained by dividing the acceptor signal (665 nm) by the donor signal (620 nm) and multiplying this value by 10,000. The 10,000-multiplying factor is used solely for the purpose of easier data handling.

#### Data Analysis

Data were then analyzed using Prism 6 (GraphPad Software, Inc., San Diego, CA, United States). *K_D_* values were obtained from saturation curves of the specific binding. Specific binding was determined by subtracting the non-specific HTRF ratio from the total HTRF ratio. *K_D_* and *B*_max_ values in saturations experiments were calculated assuming one binding site in monomeric receptor. Unlike in radioligand binding assays, *B*_max_ values obtained from HTRF data do not reflect absolute values of receptor binding sites; they are, however, useful for comparison purposes. Finally, *K_i_* values were determined according to the Cheng and Prusoff equation (Cheng, 2001). Signal-to-background (S/B ratio) calculations were performed by dividing the mean of the maximum value (μ_max_) by that of the minimum value (μ_min_) obtained from the sigmoid fits.

### cAMP Determination

Two hours before initiating the experiment, growth medium was replaced by serum-free DMEM. Then, HEK-293T cells transiently expressing CB_2_R or GPR55 were detached and resuspended in growing medium containing 50 μM zardaverine and plated in 384-well microplates (2,500 cells/well), pretreated (15 min) with the corresponding antagonists—or vehicle—and stimulated with agonists (15 min) before adding 0.5 μM forskolin or vehicle. Readings were performed after 15 min of incubation at 25°C. HTRF measures were performed using the Lance Ultra cAMP kit (PerkinElmer, Waltham, MA, United States). Fluorescence at 665 nm was analyzed on a PHERAstar Flagship microplate reader equipped with an HTRF optical module (BMG Lab Technologies, Offenburg, Germany).

### ERK1/2 Phosphorylation

To determine ERK1/2 phosphorylation, 40,000 HEK-293T-CB_2_R cells/well were plated in transparent Deltalab 96-well microplates and kept at the incubator for 24 h. Two to four hours before the experiment, the medium was replaced by serum-free DMEM. Then, cells were treated with 100 nM JWH133 and increasing concentrations of CBD in serum-free medium at 25°C for 7 min. Cells were then washed twice with cold PBS before addition of lysis buffer (20 min treatment). Ten microliters of each supernatant were placed in white ProxiPlate 384-well microplates and ERK1/2 phosphorylation was determined using AlphaScreen^®^SureFire^®^ kit (PerkinElmer) following the instructions of the supplier and using an EnSpire^®^ Multimode Plate Reader (PerkinElmer, Waltham, MA, United States).

### Statistical Analysis

The data in graphs are the mean ± SEM. Statistical analysis was performed with SPSS 18.0 software. The test of Kolmogorov–Smirnov with the correction of Lilliefors was used to evaluate normal distribution and the test of Levene to evaluate the homogeneity of variance. Significance was analyzed by one-way ANOVA, followed by Bonferroni’s multiple comparison *post hoc* test. Significant differences were considered when *p* < 0.05.

## Results

### Radioligand-Based Assays of Agonist Binding to Human CB_2_R Expressed in Isolated Membranes

**FIGURE 1:**
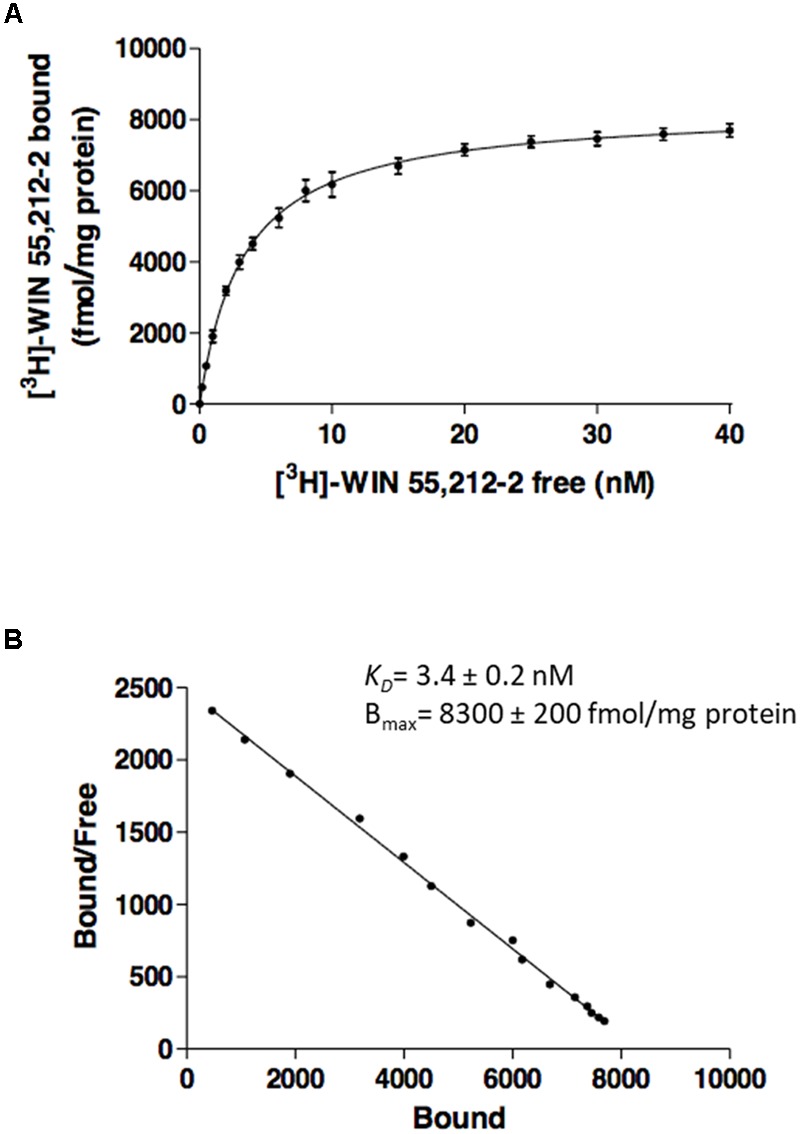
Saturation curves from radioligand binding. Saturation curve **(A)** and relative Scatchard plot **(B)** of [^3^H]-WIN 55,212-2 binding on membranes from CHO cells stably expressing human CB_2_R. Data are expressed as the mean ± SEM of three independent experiments performed in duplicate.

The effect of CBD on agonist binding to CB_2_R was first tested using a classical radioligand-binding assay and membranes isolated from CHO cells stably expressing human CB_2_R and incubated with [^3^H]-WIN 55,212-2. Data obtained from binding isotherms using increasing WIN 55,212-2 concentrations lead to a monophasic saturation curve with a *K_D_* value of 3.4 ± 0.2 nM (**Figures 1A,B**), which fits with the values reported in the literature (McPartland et al., 2009). Kinetic experiments of association of [^3^H]-WIN 55,212-2 (Supplementary Figure 1A) and its dissociation by excess unlabeled WIN 55,212-2 (Supplementary Figure 1B) showed *k*_on_ and *k*_off_ values whose quotient provides an equilibrium constant that is in agreement with the *K_D_* value calculated from saturation data.

### Competition and Saturation Assays in the Presence of CBD

**FIGURE 2:**
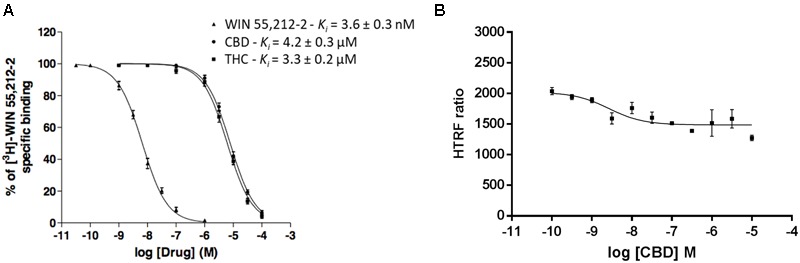
Competition curves in radioligand- and HTRF-based assays. **(A)** Competition curves of specific 3 nM [^3^H]-WIN 55,212-2 binding by unlabeled (0–10 μM) WIN 55,212-2, CBD and THC. **(B)** Competition curve of specific binding of 100 nM red CB_2_R ligand with increasing concentrations of CBD (0–10 μM) measured by HTRF. Data represent the mean ± SEM of a representative experiment (*n* = 4). HTRF ratio = (665 nm acceptor signal/620 nm donor signal) × 10,000.

Competition of 3 nM [^3^H]-WIN 55,212-2 with increasing concentrations of the “cold” compound led to a *K_i_* value of 3.6 ± 0.3 nM, well in agreement with the *K_D_* (**Figure 2A**). The *K_i_* value, when CBD was used as competitor, was in the micromolar range (4.2 ± 0.3 μM). The same phenomenon was observed with increasing concentrations of THC, *K_i_* value of 3.3 ± 0.2 μM (**Figure 2A**). Competition curves in radioligand binding assays were clearly monophasic, i.e., no significant effect was observed at submicromolar CBD concentrations.

**FIGURE 3:**
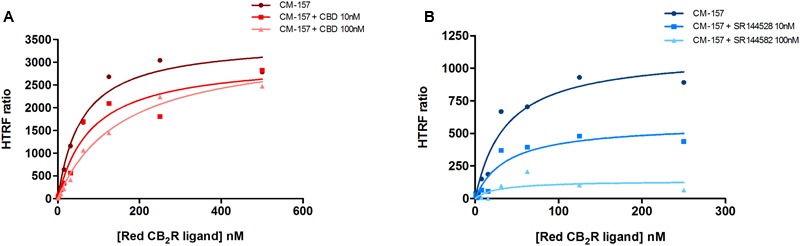
Saturation curves measured by HTRF. Saturation binding experiments of fluorescent CB_2_R ligand (CM-157) in HEK-293T cells transiently transfected with SNAP-CB_2_R in the absence or presence of 10 nM (squares) or 100 nM (triangles) CBD **(A)**, or in the absence or presence of 10 nM (squares) or 100 nM (triangles) SR144528 **(B)**. Specific binding signals were calculated by subtracting the non-specific signal determined in the presence of 100 μM unlabeled CB_2_R ligand. Data represent the mean ± SEM of a representative experiment (*n* = 4). HTRF ratio = (665 nm acceptor signal/620 nm donor signal) × 10,000.

Similar competition experiments were performed using 100 nM of a fluorescent CB_2_R agonist and a recently described homogeneous non-radioactive method performed in living cells expressing the human version of the receptor (details in Martínez-Pinilla et al., 2016). Competition of binding to the orthosteric center of the fluorescent agonist by CBD was partial but consistent, occurring at low nanomolar CBD concentrations (circa 25% reduction and IC_50_ in the 2–8 nM range) (**Figure 2B**). In order to obtain more insight on the nature of CBD binding to CB_2_R, saturation experiments were performed in HEK-293T cells expressing SNAP-CB_2_R and using increasing concentrations of red CB_2_R ligand in the presence of 10 or 100 nM CBD. Data showed that CBD decreases the affinity of the orthosteric CB_2_R ligand, whereas maximum binding (*B*_max_) was not modified (**Figure 3A** and **Table 1**). For comparison, a similar experiment was performed using 10 and 100 nM concentrations of SR144528, a CB_2_R antagonist that reportedly binds to the orthosteric site (Rinaldi-Carmona et al., 1998; Griffin et al., 1999). The *K_D_* value (**Figure 3B**) did not change when this selective CB_2_R antagonist was used. Interestingly, the antagonist reduced *B*_max_ values thus suggesting that it behaves, in binding to living cells, in an irreversible-like fashion. Taken together, the results were suggestive of CBD binding to an allosteric site at nanomolar concentrations while requiring higher concentrations (in the micromolar range) to bind to the orthosteric site.

**Table 1:** Red CB_2_R ligand (CM-157) binding parameters, in the absence and presence of CBD. Data represent the mean ± SEM of five independent experiments performed in quadruplicate.

Condition	*K_D_* (nM)	*B*_max_ (RU)^a,b^
Red CB_2_R ligand	60 ± 10	3,480 ± 210
Red CB_2_R ligand + 10 nM CBD	82 ± 28	3,060 ± 360
Red CB_2_R ligand + 100 nM CBD	160 ± 20^∗^	3,380 ± 210

*^a^RU, relative unit. ^b^*B*_*max*_ parameter does not indicate the actual level of binding sites, but it may be used for comparative purposes. ^∗^Significant differences analyzed by one-way ANOVA and Bonferroni’s multiple comparison *post hoc* tests (^∗^*p* < 0.05 *versus* absence of CBD).*

### CBD Modulates CB_2_R Signaling

**FIGURE 4:**
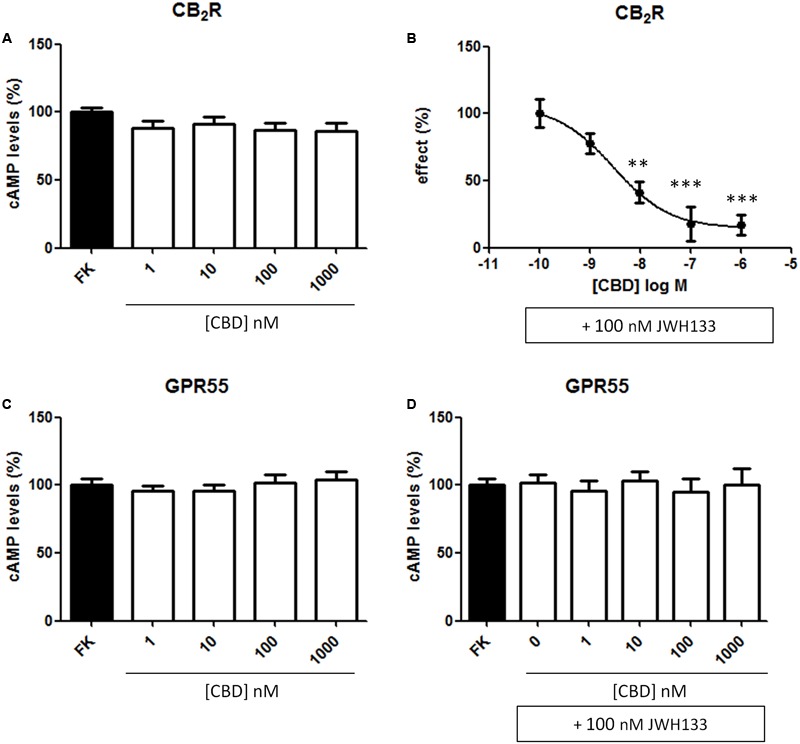
Dose-response effect of CBD on cAMP levels. HEK-293T cells were transiently transfected with cDNA encoding for human CB_2_ **(A,B)** or GPR55 **(C,D)** receptors. The effect of CBD on the decrease by JWH133 of forskolin-induced cAMP levels is displayed as a dose-response curve **(B)**. Cells were treated (15 min) with different concentrations of CBD in the absence or presence of 100 nM JWH133, a selective CB_2_R agonist and, finally, with 0.5 μM forskolin (15 min). Agonist-induced reduction in cAMP was 31 ± 4 and data (mean ± SEM) are given in percentage of the 500 nM forskolin-induced cAMP concentration. Significant differences were analyzed on data from seven different experiments; one-way ANOVA and Bonferroni’s multiple comparison *post hoc* test were used for statistical analysis (*F*_4,71_ = 15, *p* < 0.001) (^∗∗^*p* < 0.01, ^∗∗∗^*p* < 0.001, *versus* 0 nM CBD treatment).

**FIGURE 5:**
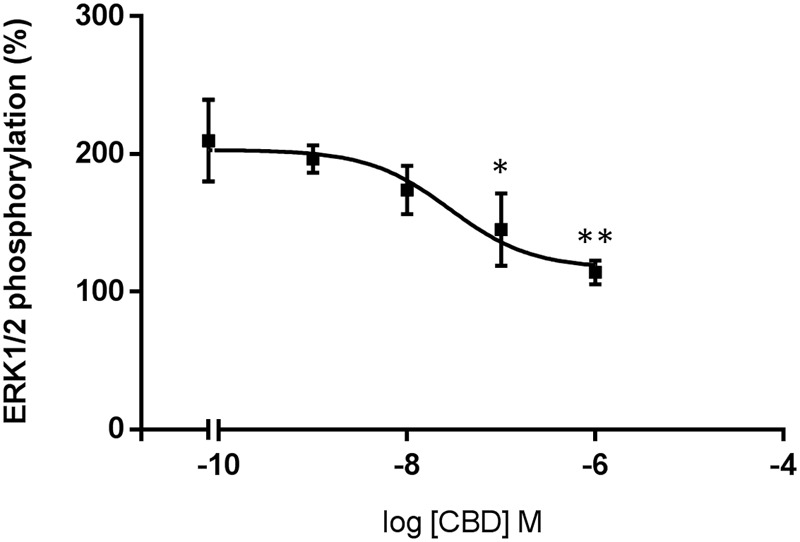
Dose-response effect of CBD on ERK1/2 phosphorylation. HEK-293T cells transfected with human CB_2_R were stimulated with 100 nM of JWH133 agonist in the presence of CBD. ERK1/2 phosphorylation levels were determined by using AlphaScreen^®^SureFire^®^ kit. Data are normalized and expressed as a percentage of over-basal response (mean ± SEM). Significant differences were analyzed on data from three different experiments; one-way ANOVA and Bonferroni’s multiple comparison *post hoc* test were used for statistical analysis (*F*_4,10_ = 11, *p* < 0.01) (^∗^*p* < 0.05, ^∗∗^*p* < 0.01, *versus* 0 nM CBD treatment).

CBD action has been often associated to CB_2_R expression but its binding to the orthosteric site of the receptor is seemingly unspecific; we hypothesized that it might act as allosteric modulator. To test the hypothesis, we first confirmed that the compound (concentration range: 1–1,000 nM) did not significantly engage any G-protein-coupled signal in HEK-293T cells expressing the human version of CB_2_R (**Figure 4A**). CBD was also tested in cells expressing the human version of GPR55, a GPCR that may bind cannabinoids, and the results were similar to those in cells expressing the CB_2_R (**Figure 4C**). We next tested whether CBD could affect the action of the selective CB_2_R agonist, JWH133, on forskolin-induced intracellular cAMP levels. The results show that the decrease in cAMP levels induced by 100 nM JWH133 was dose-dependently blocked by CBD (*F*_4,71_ = 15, *p* < 0.001) (**Figure 4B**). IC_50_ of the CBD effect was 3 ± 0.4 nM. Again, the response of HEK-293T transiently transfected with GPR55 to the combined treatment with CBD and JWH133 was not significantly different than that achieved by CBD alone (**Figure 4D**). Similar experiments were undertaken but using ERK1/2 phosphorylation as a read-out. The results in **Figure 5** show that significant ERK1/2 phosphorylation triggered by 100 nM JWH133 was dose-dependently blocked by CBD (*F*_4,10_ = 11, *p* < 0.01) with an IC_50_ of 29 ± 0.3 nM. These signaling data showed that CBD was able to negatively modulate CB_2_R signaling at concentrations lower to those required to significantly bind to the orthosteric center of the receptor.

## Discussion

The results reported confirm that nanomolar CBD concentrations are not able to displace the binding of WIN 55,212-2 to the orthosteric center of the CB_2_R in radioligand binding assays. The binding data using the non-radioactive homogeneous method performed in living cells showed, however, that the binding of the fluorescent orthosteric ligand to HEK-293T cells expressing CB_2_R was slightly but consistently modified by nanomolar concentrations of CBD. The most plausible interpretation of these data is an allosteric effect disclosed in HTRF-mediated binding assays and a very small difference in the binding mode of radiolabeled WIN 55,212-2 or fluorescence-labeled CM-157 to CB_2_R. Unfortunately, WIN 55,212-2 could not be used in non-radioactive assays as it non-specifically interacted (for unknown reasons) with the HTRF probes. We found the same non-specific interaction with the fluorescent labeled CBD (data not shown). Structural differences related to the binding of different agonists that may be revealed by HTRF have been discussed elsewhere (Martínez-Pinilla et al., 2016). In that paper, the extensively studied ligand AM630 displayed a biphasic curve on competing with CM-157 binding to CB_2_R. The results here presented could fit with CBD binding to the high-affinity sites disclosed using AM630 but not to the low-affinity ones. Alternatively, AM630 reduction of high-affinity binding, which occurred at subnanomolar concentrations of AM630, could be due to the same mechanism than that of CBD. In fact, the two compounds displayed on HTRF-based binding assays two components, one at low concentrations, and another at higher concentrations.

A further piece of information from results using the selective orthosteric CB_2_R antagonist, SR144528, was the irreversible-like behavior when performing HTRF-based binding to living cells. In binding or functional assays using isolated membranes or tissue extracts from CB_2_R-expressing cells, the antagonist acts in a reversible fashion (Rinaldi-Carmona et al., 1998; Griffin et al., 1999). CBD seemingly allosteric action was detectable by functional experiments (cAMP and pERK1/2 assays) in which we show that the effect of a selective CB_2_R agonist was modulated by CBD at physiologically relevant (nanomolar) concentrations. The participation of a third component, acting as mediator of CBD effects on CB_2_R signaling cannot be ruled out.

In the absence of consensus data showing a direct interaction between CBD and cannabinoid receptors, only indirect evidence suggests that CBD could be a modulator of endocannabinoid signaling. Accordingly, CBD was suspected to act as allosteric modulator of cannabinoid receptors (Rimoldi and Bow, 2016). In agreement with this possibility, Laprairie et al. (2015) were the first to suggest that CBD acts as allosteric modulator of CB_1_R; although no binding studies were performed, the allosteric site in the receptor was mapped to two cysteine residues in the N-terminal end. Therefore, CBD reduces both potency and efficacy of endogenous and exogenous cannabinoids on ERK1/2-PLCβ3-dependent signaling in an heterologous expression system, and in cells endogenously expressing the receptor. Authors also reported that CBD affects the kinetics of β-arrestin recruitment and CB_1_R internalization.

Allosteric modulators of natural origin do usually provide negative modulation in both enzymology and pharmacology. Allosteric action was negative in both the results reported by Laprairie et al. (2015) on CB_1_R-mediated signaling and ours on CB_2_R-mediated signaling; the allosteric effect in both cases seems to be, at least in part, mediated by a CBD-induced decrease in affinity of the orthosteric agonist (**Table 1**). One interesting possibility would be that cannabinoids may produce effect of allosteric nature on a variety of GPCRs. In support of this option, Lane et al. (2010) showed that the endocannabinoid 2-AG acts as an allosteric modulator of the human adenosine A_3_ receptor. Adenosine A_3_ receptors are coupled to a heterotrimeric G_i_ protein and the action of 2-AG resulted in a decrease in the potency of agonists and in the basal signaling of this adenosine receptor subtype. Their negative effects on the receptor-mediated cAMP response are similar to those reported here for CBD acting on CB_2_R. CBD is also described as an allosteric modulator of μ- and ∂-opioid receptors (Vaysse et al., 1987; Kathmann et al., 2006). Both Δ^9^-THC and CBD accelerate the dissociation of opioids from the receptors although the function of CBD on opioid receptors was not studied (Kathmann et al., 2006).

Ligand-gated receptors are also affected by CBD. Indeed, CBD inhibits currents mediated by serotonin 5-HT_3A_ receptors expressed in *Xenopus laevis* oocytes (Yang et al., 2010). Higher CBD concentrations, in the micromolar range, are able to allosterically modulate ligand-gated glycine receptors impacting on the role of glycine in postsynaptic transmission in the adult spinal cord (Ahrens et al., 2009; Foadi et al., 2010). The interacting motif is mapped to Ser^276^ of the alpha1 subunit of the receptor (Foadi et al., 2010), thus seemingly different from the binding motif reported for CB_1_R (Laprairie et al., 2015). Homologous residues to those in the CB_1_R sequence are not present in the CB_2_R one and, therefore, the putative binding site may not be located in the N-terminal domain of the CB_2_R; in fact, the putative N-terminal domain is much shorter for CB_2_ than for CB_1_ receptors (33 *versus* 116 amino acids^2^). Elucidation of the structure for the CB_1_R rises hope for a similar achievement for CB_2_R and, subsequently, for detecting allosteric sites that would help in designing novel drug discovery approaches targeting cannabinoid receptors. The N-terminal domain of the A_3_ receptor is also too short to be involved in the mode of action of 2-AG. Human μ and ∂ opioid receptors have longer N-terminal domains, 68 and 47, respectively, but there is no obvious homology between them. The two cysteine residues present in the N-terminal end of CB_1_R and that putatively conform a CBD binding site are not present in the N-terminal domain of ∂-opioid receptors. In summary, further experimental effort is needed to identify common motives for endocannabinoid action on ligand-gated and GPCRs, or to identify molecules interacting with these receptors and acting as mediators of the allosteric-like effect disclosed by CBD. Finding CBD binding motives in cannabinoid receptors or non-GPCR CBD targets would help in understanding some of the actions reported for endocannabinoids and for natural cannabinoids such as THC and CBD.

GPCRs constitute the target of 40–45% of current medicines that act, as agonists or antagonists, via the orthosteric center. The discovery of GPCR allosteric modulators or of modulators of signaling at concentrations 1–2 orders of magnitude below the IC_50_ values obtained in competing with the binding of orthosteric compounds, opens new perspectives for therapeutic benefit.

## Author Contributions

EM-P designed and executed HTRF experiments, analyzed HTRF data, and edited the manuscript; IR-R, EA, and GN cloned the fusion proteins and performed HTRF and cAMP assays; JL directed the pharmacological assays in CIMA and contributed to scientific discussions and editing the manuscript. JO participated in the design and the synthesis of labeled compounds for HTRF assays. EC contributed to the analysis of results. CF-V and XN contributed to the conception and design of the study and were implied in the process of writing the manuscript, the analysis and interpretation of data, and critically reviewed and approved the manuscript; KV and FV designed and supervised the radioligand binding experiments and contributed to manuscript edition; KV and PB performed the radioligand binding assays; RF contributed in formulating the initial hypothesis, directed the work in the University of Barcelona (Barcelona), coordinated efforts from different laboratories, and participated in writing the manuscript.

## Conflict of Interest Statement

The authors declare that the research was conducted in the absence of any commercial or financial relationships that could be construed as a potential conflict of interest.

## Footnotes

1 www.guidetopharmacology.org

2 www.uniprot.org

## Abbreviations

AEA: anandamide
2-AG: 2-arachidonyl glycerol
CBD: cannabidiol
CB_1_R: cannabinoid receptor subtype 1
CB_2_R: cannabinoid receptor subtype 2
CNS: central nervous system
GPCR: G-protein-coupled-receptor
HTRF: homogenous time-resolved fluorescence resonance energy transfer
Δ^9^-THC: Δ^9^-tetrahydrocannabinol
TR-FRET: time-resolved fluorescence resonance energy transfer
