# Supplementary material for: Binding and Signaling Studies Disclose a Potential Allosteric Site for Cannabidiol in Cannabinoid CB2 Receptors
Source: Front Pharmacol. 2017 Oct 23;8:744. doi: 10.3389/fphar.2017.00744 (PMC5660261; doi:10.3389/fphar.2017.00744)
Supplement: Supplementary file 1 [file Image_1.PDF]

Supplementary Figure 1.

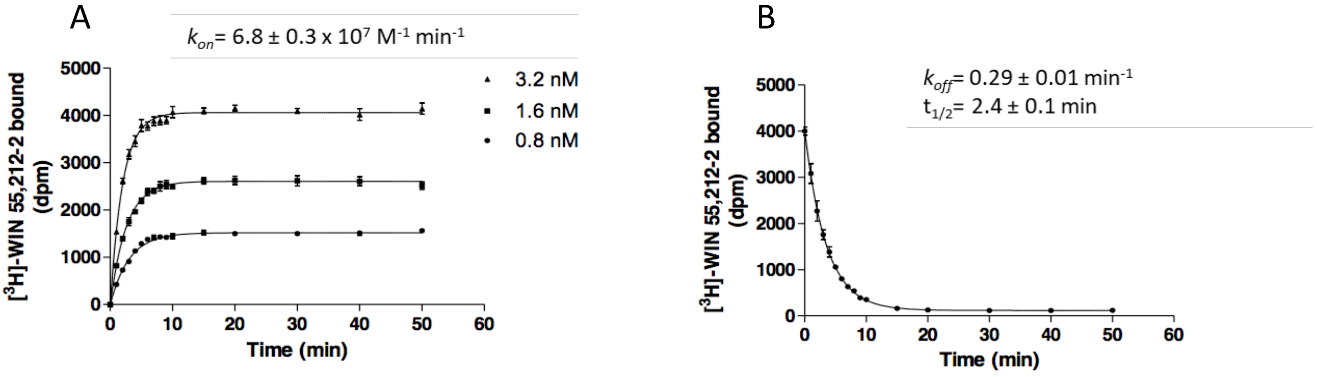

Supplementary Figure 1. Radioligand binding kinetic assays showing association and dissociation of [^{3}H]-WIN 55,212-2. Association curves of three different concentrations (0.8, 1.6 and 3.2 nM) of [^{3}H]-WIN 55,212-2 (A) and dissociation curve of 3.2 nM [^{3}H]-WIN 55,212-2 using 10 µM unlabeled WIN 55,212-2 (B). Data are expressed as the mean ± SEM of three independent experiments performed in duplicate.
